# A novel Fas ligand plays an important role in cell apoptosis of *Crassostrea hongkongensis*: molecular cloning, expression profiles and functional identification of *Ch*FasL

**DOI:** 10.3389/fimmu.2023.1267772

**Published:** 2023-10-05

**Authors:** Yanping Qin, Weitao Wan, Jiangwei Li, Zhongyu Wang, Yue Yang, Jun Li, Haitao Ma, Ziniu Yu, Zhiming Xiang, Yuehuan Zhang

**Affiliations:** ^1^ Key Laboratory of Tropical Marine Bio-resources and Ecology, Guangdong Provincial Key Laboratory of Applied Marine Biology, South China Sea Institute of Oceanology, Chinese Academy of Sciences, Guangzhou, China; ^2^ Hainan Provincial Key Laboratory of Tropical Marine Biology Technology, Sanya Research Institute of Marine Ecological Environment Engineering, Tropical Marine Biological Research Station in Hainan, Chinese Academy of Sciences, Sanya, China; ^3^ Innovation Academy of South China Sea Ecology and Environmental Engineering, Chinese Academy of Sciences, Guangzhou, China; ^4^ University of Chinese Academy of Sciences, Beijing, China; ^5^ College of Animal Science and Technology, Guangxi University, Nanning, China

**Keywords:** *Crassostrea hongkongensis*, Fas Ligand, relative expression, functional verification, apoptosis

## Abstract

**Background:**

Apoptosis regulates normal development, homeostasis, immune tolerance and response to environmental stress by eliminating unwanted or diseased cells, and plays a key role in non-specific immunity of invertebrates. The exogenous pathway mediated by death receptors and death ligands is a very important pathway for cell apoptosis. Death ligands are mainly members of the tumour necrosis factor (TNF) family, of which FasL is an important member. The deep involvement of FasL in vertebrates cell apoptosis and immunity has been reported many times, but there is limited research on the FasL gene in shellfish, and its functional importance in oyster cell apoptosis and immunity remains unclear.

**Methods:**

The full length of *Ch*FasL was identified and cloned based on the genome of *Crassostrea hongkongensis*. Quantitative PCR was used to detect the relative expression of *Ch*FasL in different developmental stages and tissues, as well as the changes of relative expression in hemocytes after bacterial infection. The expression position of *Ch*FasL in HEK293T cells was also located by subcellular localization, and the effect of increased recombinant protein content on the activity of reporter genes p53 and p21 was studied by dual-fluorescence reporter gene. Finally, the changes of apoptosis rate in hemocytes after *Ch*FasL silencing was identified by RNA interference technology.

**Results:**

We identified a novel FasL gene from *C. hongkongensis* and named it *Ch*FasL. We found that *Ch*FasL has potential N-linked glycosylation site, a transmembrane domain and a TNF region, which was a typical characteristics of TNF family. *Ch*FasL was expressed in all developmental stages of larvae and in all tissues of oysters. After stimulation by *V. alginolyticus* or *S. haemol*yticus, its relative expression in hemocytes increased significantly, suggesting that *Ch*FasL was deeply engaged in the immune response process of *C. hongkongensis* to external microbial stimulation. The results of subcellular localization showed that *Ch*FasL was mainly distributed in the cytoplasm of HEK293T cells. With the overexpression of the recombinant protein pcDNA3 1- *Ch*FasL, the activity of p53 and p21 significantly increased, showing a positive regulatory effect. Moreover, after dsRNA successfully reduced the relative expression of *Ch*FasL, the apoptosis rate of hemocytes was significantly lower than that the dsGFP group.

**Conclusion:**

These results comprehensively confirmed the important role of *Ch*FasL in the apoptosis process of *C. hongkongensis*, which provided the basis and premise for the in-depth understanding of the immune function of apoptosis in molluscs, and also contributed to the research on the pathogenic death mechanism and disease resistance breeding of marine bivalves.

## 1 Introduction

Apoptosis is a conservative, gene directed process used to eliminate unnecessary or dangerous cells without disrupting tissue biological functions. It plays an important role in embryonic development, cell proliferation, especially in maintaining body homeostasis (1–3). This physiological process is one of the major immune responses of the host defense system to the chemical stress of eukaryotic cells, and is related to several pathogenic infections. So, apoptosis plays a key role in the innate immune system (4, 5). In 1972, Kerr JF, Wyllie AH and Currie AR first described and defined the morphological hallmarks of cell apoptosis (2). Afterwards, a series of physiological function studies on apoptosis were identified, and Brenner S, Horvitz HR and Sulston JE were awarded the Noble Prize in 2002 for systematically describing the mechanisms of programmed cell death (apoptosis) (6–8).

In mammals, cell apoptosis can be transduced and function via exogenous pathway or endogenous pathway (9). The exogenous pathway starts with the binding of death ligands and death receptors. Chemical agents and pathogenic infections can activate the extrinsic pathway via death receptors (5, 6). In human cells, many genes and factors are involved in the death receptor superfamily, including FasL, TNF-R1, DR3 (Apo-3, WSL-1), DR-4 (TRAIL-R1), DR-5 (TRAIL-R2) and EDA-R (ectodermal dysplasia receptor) (10–15). Fas ligand (FasL) and Fas system play a vital role in the death receptor superfamily pathway. The combination of Fas and FasL induces the aggregation of Fas. Then these activated Fas polymers recruit Fas-associated proteins with Death domain and caspase-8 proenzyme to form the death induction signaling complex via Fas-associated protein with Death domain. Activated caspase-8 directly or indirectly recruits and activates caspase-3, leading to cell apoptosis (11, 14, 16–20). In recent years, FasL and Fas system have been well characterized in many vertebrates and invertebrates (2, 11, 16, 21). A FasL-like molecule in leucocytes of the gilthead seabream was identified by Alberto Cuesta and his group (22). In addition, they pointed out that resting leucocytes express cytosolic FasL-like, rather than membranous. Moreover, it has been confirmed that soluble form of FasL is an important mediator of nonspecific cytotoxic cells of innate immunity of teleost (22, 23). Tomofumi Kurobe and his group isolated and sequenced FasL cDNA from Japanese flounder, *Paralichthys olivaceus.* They demonstrated that fish possess the FasL system (24). A novel FasL of the Tumor Necrosis Factor (TNF) superfamily has been demonstrated in mollusk abalone and has been shown to play an important role in accelerating cell apoptosis and maintaining the homeostasis of immune responses (11). Although FasL has been well characterized in many vertebrates and invertebrates, except for a novel FasL found in mollusk abalone, there is little information about FasL homologues in mollusks.

*Crassostrea hongkongensis* is a very important economic species of shellfish in Southern China, mainly distributed in low-salt estuaries. They often face stress stimuli and immune challenges, such as sudden changes in water salinity and temperature, as well as microbial infections, which often lead to large-scale death and serious economic losses (25). Therefore, it is urgent to study the immune response process of *C. hongkongensis*. Similar to other shellfish, Hong Kong oyster has no specific immunity and only relies on innate immunity to resist external environmental stresses, including changes in environmental conditions and pathogenic infection. Apoptosis is an important process to maintain immune homeostasis. As we know, FasL play a key role in the initiation of cell apoptosis (11, 15), so the study of FasL in *C. hongko*ngensis is helpful to further understand the process of immune apoptosis and reduce abnormal death caused by microbial infections.

In this study, the full-length cDNA of FasL was identified and characterized from *C. hongkongensis*, and named *Ch*FasL. We investigated the relative expression profiles of *Ch*FasL in different tissues and developmental stages. Additionally, we also studied the spatio-temporal expression of *Ch*FasL after challenge with bacterial pathogen, this would help us to understand the role of FasL in the innate immunity system of *C. hongkongensis*. Furthermore, we also analyzed the subcellular localization and transcriptional activity regulation of *Ch*FasL.

## 2 Materials and methods

### 2.1 Molecular cloning and analysis of ChFasL

The partial sequence of *Ch*FasL was identified from *C. hongkongensis* genome database and confirmed that the gene was annotated as an uncharacterized and not studied during BLASTed in NCBI. Then, gene-specific primers were designed to clone the sequences of *Ch*FasL based on partial sequence. Polymerase chain reaction (PCR) at 50 μL, including 1 μL of each primer, 0.5 μL of ExTaq polymerase, 5 μL of 10×ExTaq buffer, 0.25 mM dNTPs, and 1 μg of cDNA. PCR was performed for 30 cycles at 95 °C for 50 s, 57 °C for 50 s, 72 °C for 1 min and a final extension step at 72 °C for 10 min. PCR products were tested on 1% agarose gel, then the expected size was gel extracted (QIAquick gel extraction kit, Magen), subcloned into a pMD19T cloning kit (TaKaRa, Japan), transformed into competent cells of *Escherichia coli* and monoclone was selected for sequencing. Based on the genetic sequences identified above, 3’ and 5’ ends primers were designed for rapid amplification of cDNA end sequences (RACE) (**Table 1**), as well as SMART-RACE™ kit (TAKARA, Japan) was used according to the manufacturer’s instructions. Based on the outer primer and inner primer, nested PCR was performed. Eventually, the full length cDNA of *Ch*FasL was successfully obtained.

**Table 1:** Primers used in this study.

Primer	Sequence (5’→ 3’)	Comment
*Ch*FasL-F1	ATGGTTTCCTGTGCACCAAAGACTTG	ORF amplification
*Ch*FasL -R1	CAGCTTAAACAATCCGATGTAGTTATT	
*Ch*FasL -F2	AAAGGACACTGCCATTCAAGA	3’ Race
*Ch*FasL -F3	TAGACCTCGTGTTTAGTAAGTTTGG	
*Ch*FasL -R2	ATCCTTAGTAAATGCCGTGCC	5’ Race
*Ch*FasL -R3	ACTGAACTCTTTAGGTCCCCG	
*Ch*FasL -F4	TACCGGACTCAGATCTCGAGATGGTTTCCTGTGCACC	pEGFP-N1- *Ch*Cas 3
*Ch*FasL -R4	TGGTGGCGACCGGTGGATCCCAGCTTAAACAATCCG	
*Ch*FasL -F5	AGTGTGGTGGAATTCATGGTTTCCTGTGCACCAAAG	pcDNA 3.1- *Ch*Cas 3
*Ch*FasL -R5	CCCTCTAGACTCGAGCAGCTTAAACAATCCGATGT	
*Ch*FasL -F6	GGATCCTAATACGACTCACTATAGGGAAACCGTTGAAAATGAGG	RNAi primers qPCR primers
*Ch*FasL -R6 *Ch*FasL -F7 *Ch*FasL -R7	GGATCCTAATACGACTCACTATAGGCTTAGTAAATGCCGTGCC AGGAAACCGTTGAAAATGAGGA AACTGAACTCTTTAGGTCCCCG	
EFlα-F	AAGATATTGCAGCTTTAGTCGT	
EFlα-R	TTCTGTCCCATACCAACCAT	

The characteristics of *Ch*FasL were analyzed with SMS (http://www.bio-soft.net/), PROSITE (https://prosite.expasy.org/) and SMART (http://smart.embl-heidelberg.de/smart). Phylogenetic analyses and homologous evolutionary analysis of *Ch*FasL sequences were conducted using MEGA 5.1 and DNASTAR software, respectively.

### 2.2 Tissues collection and bacterial challenge

The oysters used in the experiment were artificially cultivated in an open circulating culture system and fed with sufficient food. Prior to the experiment, all oysters used were temporary cultivated for one week. Then, 5 healthy and active oysters were selected for the experiment, and 8 tissues were sampled, including hemocytes, gills, mantle, adductor muscle, labial palps, heart, gonads and digestive gland, to compare the differential expressions of *Ch*FasL. The fertilized eggs, cleavage, morula, blastula, gastrula, trochophore and D-larvae were sampled at the reproductive stage.

The bacterial challenge experiment was as follow: a total of 135 oysters with similar size were evently divided into 3 groups (2 experimental groups and one control group). *staphylococcus haemolyticus* and *Vibrio alginolyticus* were separately resuspended in 0.1 M phosphate-buffered saline (PBS) at 1.0×10^9^ cells/L. Then, 100 μL of bacterial suspension or PBS was injected into the adductor muscle of *C. hongkongensis*. Haemolymph was collected from 5 oyster individuals at 0 h, 3 h, 6 h, 12 h, 24 h, 48 h and 72 h after injection. The haemolymph was immediately centrifuged and then supernatant was removed and precipitated hemocytes were collected, preserved in TRIzol (Invitrogen, USA), and stored at -80 °C for RNA extraction.

### 2.3 Isolation of total RNA and cDNA synthesis

Total RNA was extracted from collected samples using TRIzol method according to the operation instructions. RNA concentrations, integrity and purity were detected using a Nanodrop 2000c spectrophotometer (Thermo, USA) and 1.5% agarose gels. The concentration of the RNA was diluted up to 1 mg/mL before cDNA synthesis. cDNA synthesis was carried out according to a PrimeScript RT reagent kit with gDNA Eraser (TaKaRa, Japan). Firstly, gDNA Eraser was used for removing DNA from the total RNA (1 μg), and then the reverse transcription reaction was performed by using the reverse transcription enzyme and the reverse transcription primer mix. Finally, synthesized cDNA was diluted 20-fold and stored at 20 °C.

### 2.4 *Ch*FasL relative expressions analysis by real-time PCR

The relative expression of *Ch*FasL was analyzed by quantitative real-time PCR (qPCR) using gene-specific primers. The gene-specific primers were designed based on the ORF of *Ch*FasL, and actin and elongation of the factor 1α gene (EF1α) was selected as the house-keeping gene (**Table 1**). qPCR was performed in a Light-Cycler 480II system (Roche, USA) using a total volume of 20 μL, containing 10 μL of 2×Real Star Green Power mixture (Genestar, China), 0.50 μM of each primer, and 1 μL diluted cDNA. The PCR program was set as follows: 94°C for 5 min, 40 cycles of 94°C for 10 s, 57°C for 10 s, and 72°C for 20 s. Each run was included a blank (water), a negative control (RNA not reverse-transcripted but treated with DNase I) and a positive cDNA control. In order to ensure the specificity of PCR amplification, a single peak melting curve was ensured.

### 2.5 Construction of recombinant plasmids

In order to understand the expression and function of *Ch*FasL, the ORF of *Ch*FasL was subcloned into pEGFP-N1 and pcDNA3.1 eukaryotic expression vectors. The specific primers used to construct recombinant plasmids are listed in **Table 1**. Recombinant plasmids were obtained using a One Step Cloning Kit (Vazyme, China). Briefly, the fragment of *Ch*FasL was obtained by PCR and purified in 1.5% agarose gel. The empty plasmids were digested by 2 different restriction enzymes. Then, the purified PCR product and the digested plasmids were linked in a volume of 20 μL containing 4 μL 5×CE II buffer, 2 μL Exnase II, 200 ng purified fragments and 200 ng linear plasmid, and mixture reacted at 37 °C for 30 minutes. Finally, the mixture was transformed into *E. coli* DH5α cells. The recombinant plasmids were sequenced and confirmed that the gene sequence has been successfully constructed into the plasmid. Through the above steps, the recombinant plasmid was successfully constructed. The recombinant plasmids used for transfection were extracted an EndoFree Plasmid Mini kit (OMEGA, USA).

### 2.6 Subcellular localization

In this study, HEK293T cells were used for subcellular localization analysis of *Ch*FasL. HEK293T cells were cultured in Dulbecco’s modified Eagle’s medium (DMEM) containing 10% foetal calf serum (Gibco, USA) and 1× antibiotics (streptomycin and penicillin, Gibco) in an incubator with temperature, humidity and CO_2_ of 37 °C, 95% and 5%. According to the manufacture instructions, the plasmid was immediately transfected into the HEK293T cells with Lipofectamine 2000 (Invitrogen, USA). The mixture containing 450 μL serum-free culture medium, 2.5 μg recombinant plasmid *Ch*FasL-pEGFP-N1 and 5 μL Lipofectamine 2000 were transfected into HEK293T cells. After 10 hours of transient transfection, the culture medium was replaced by DMEM with 10% foetal calf serum and 1× antibiotics. After 48 hours of transfection, HEK293T cells were treated as follow: cells were gently washed twice with PBS solution, fixed with 4% paraformaldehyde for 10 min, and stained with 4,6-diamidino-2-phenylindole hydrochloride (DAPI) for 5 min, and then washed with PBS twice. Finally, the subcellular localization of *Ch*FasL was observed directly under a fluorescence microscope.

### 2.7 Luciferase reporter assay

When HEK293T cells plated on a 48-well plate grew to 50% confluence, the mixture of the reporter plasmid (200 ng/well), pRL-TK (20 ng/well), pcDNA3.1-*Ch*FasL (0, 200, 300 and 400 ng/well, respectively) and pcDNA3.1 (400, 200, 100 and 0 ng/well, respectively) were co-transfected into cells using Lipofectamine 2000 in a serum-free culture medium. Each group was established 3 repeats. After 48 hours of co-transfection, HEK293T cells in each plate were gently washed twice with 100 mL PBS, and then 30 mL 1× passive lysis buffer was added to the plate, which was treated at room temperature for 10 minutes. Finally, successively added 50 mL luciferase assay reagent II and 50 mL stop & glo reagent, and Firefly and Renilla luciferase activities were corresponding detected using a dual-luciferase reporter assay system (Promega, USA).

### 2.8 *Ch*FasL silencing and apoptosis detection

The primers used for silencing *Ch*FasL were designed according to the manufacturer’s instructions of the T7 RiboMAX Express RNAi system Kit (Promega, USA) and shown in **Table 1**. According to the manufacturer’s instructions of the Kit, the dsRNA used for silencing *Ch*FasL was prepared and injected into the adductor muscle of oysters also. After 96 hours of the dsRNA injection, haemolymph was collected from 5 oysters. Oyster hemocytes were treated with Annexin V-FITC apoptosis detection kit (Vazyme, China), and then the apoptosis rate was measured using flow cytometry. In brief, the cells were centrifuged at 300 g and 4 °C for 5 min to obtain a pellet of cells, then gently washed twice with pre-cooled PBS. Secondly, 100 μL of 1× binding buffer was added to resuspend the cells. Subsequently, 5 μL of Annexin V-FITC and 5 μL of PI staining solution were successively added and gently mixed. Then, the samples were incubated in dark for 15 min, 400 μL 1×binding buffer was added and gently mixed. Finally, a flow cytometry was used for detecting the apoptosis rate of the hemocytes immediately.

### 2.9 Statistical analysis

In the experiment, the mean± standard deviation (M ± SD) was used. The luciferase reporter data analysis was compared using independent-samples T test and the relative expression analysis of *Ch*FasL were compared using one-way analysis of variance followed by multiple comparison tests using SPSS18. Significant difference represents *P* < 0.05, and highly significant represents *P* < 0.01.

## 3 Results

### 3.1 cDNA cloning and molecular characteristics of *Ch*FasL

**Figure 1:**
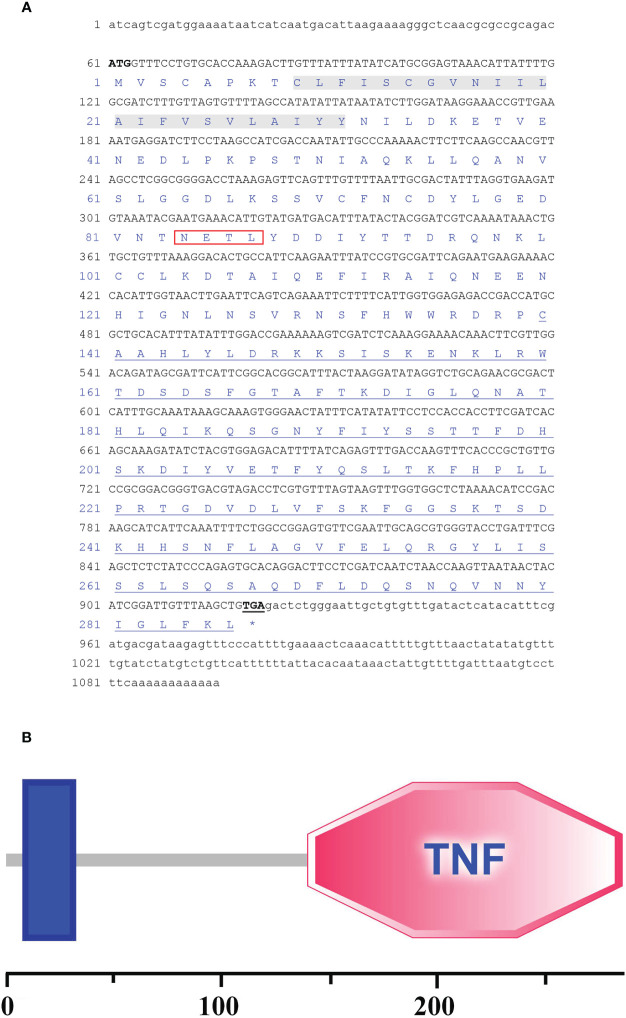
**(A)** The full-length cDNA sequence and the deduced amino acid sequences of *C. hongkongensis Ch*FasL. Nucleotides and amino acids are numbered on the left-hand side. The ORF contains a peptide signal (Red box), a transmembrane domain (Gray bottom) and a TNF domain (Underlined). **(B)** Sequence structure of *Ch*FasL.

The full-length cDNA sequence of *Ch*FasL was 1095 bp, consisting of a 60 bp 5′-untranslated region (UTR), an 861 bp Open Reading Frame (ORF) sequence, and a 174 bp 3′-UTR (GenBank accession number: OR157977) (**Figure 1A**). The above ORF was expected to encode a protein sequence containing 287 amino acids with a calculated molecular weight (Mw) of 32454.54Da and a predicted theoretical isoelectric point (pI) at 6.06 predicted by Expasy Compute pI/Mw tool (https://web.expasy.org/computepi/). The ORF of *Ch*FasL was characterized by a transmembrane region that started at position 9 and ended at position 31, and a TNF domain that was at positions 140 to 286 (**Figures 1A, B**), which was predicted by the SMART and PROSITE programs.

### 3.2 Sequence alignment and phylogenetic analysis of *Ch*FasL

**Figure 2:**
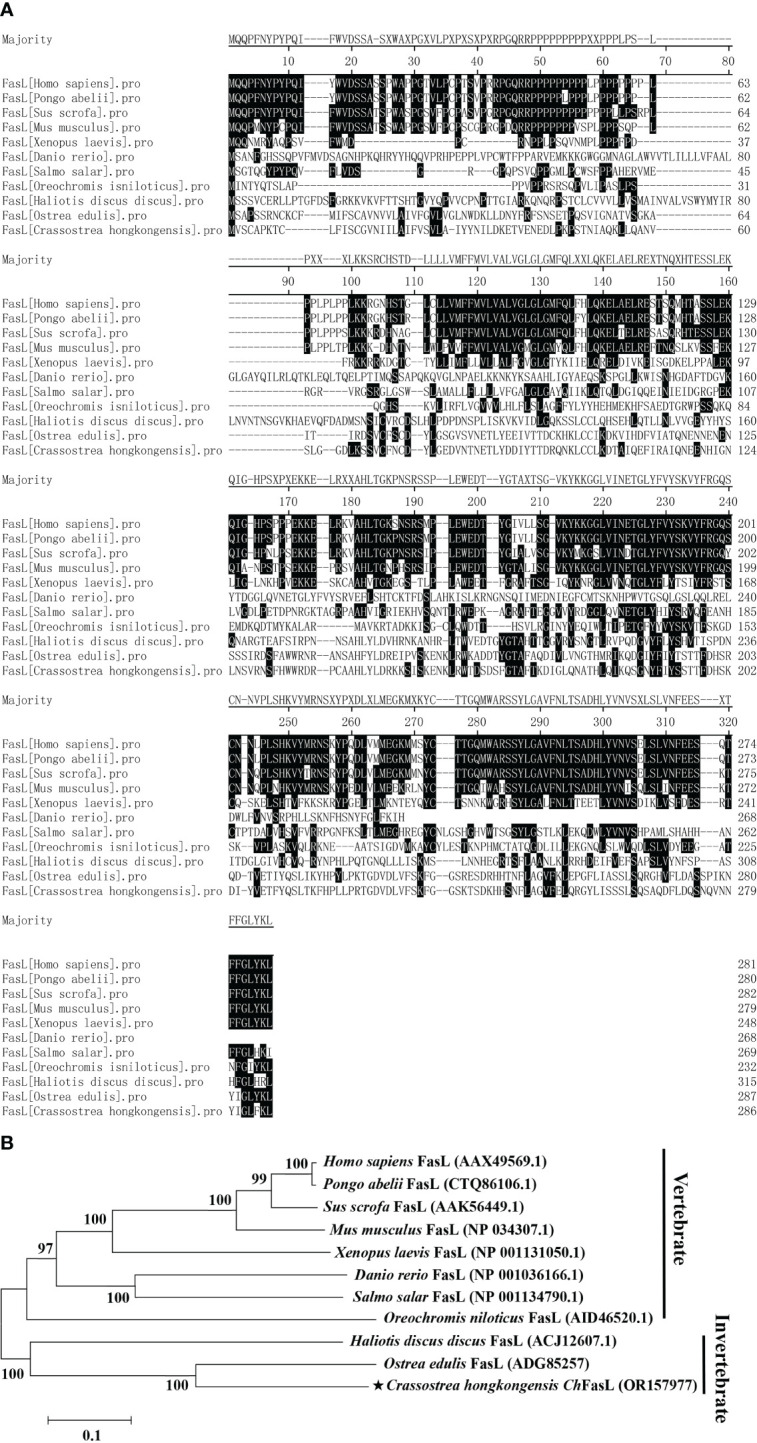
**(A)** Amino acid sequence homology alignment of FasL from *Homo sapiens* (AAX49569.1), *Pongo abelii* (CTQ86106.1), *Sus scrofa* (AAK56449.1), *Mus musculus* (NP_034307.1), *Xenopus laevis* (NP_001131050.1), *Danio rerio* (NP_001036166.1), *Salmo salar* (NP_001134790.1), *Oreochromis niloticus* (AID46520.1), *Haliotis discus discus* (ACJ12607.1), *Ostrea edulis* (ADG85257) and *Crassostrea hongkongensis Ch*FasL (OR157977). **(B)** Phylogenetic analysis of FasL from different species. *Ch*FasL is marked with “★”. Numbers at tree nodes refer to bootstrap values from 1000 replications. The Phylogenetic tree was constructed using the MEGA program on the basis of the full amino acid sequences.

Multiple sequence alignment showed that *Ch*FasL has very low similarity to other known FasL proteins from other species (**Figure 2A**). The amino acid sequences of *Ch*FasL and other FasL homologues were then used to build a clear phylogenetic tree by MEGA 5.1 software to show their evolutionary relationships. The results showed that the *Ch*FasL protein was most closely related to FasL of *Ostrea edulis* and belonged to the group of invertebrates, and had a distant evolutionary distance from other vertebrates, indicating a different ancestral origin of FasL (**Figure 2B**). There was little overlap of sequence alignment between *Ch*FasL and other FasL homologies, and the overlap mainly occurred in the transmembrane region and TNF region, especially the TNF region, while the homology of other regions was very low (**Figure 2B**). These results confirm that *Ch*FasL might be a FasL homolog and perform similar functions to other FasL proteins.

### 3.3 Relative expressions of *Ch*FasL in different tissues and in response to bacterial challenge

**Figure 3:**
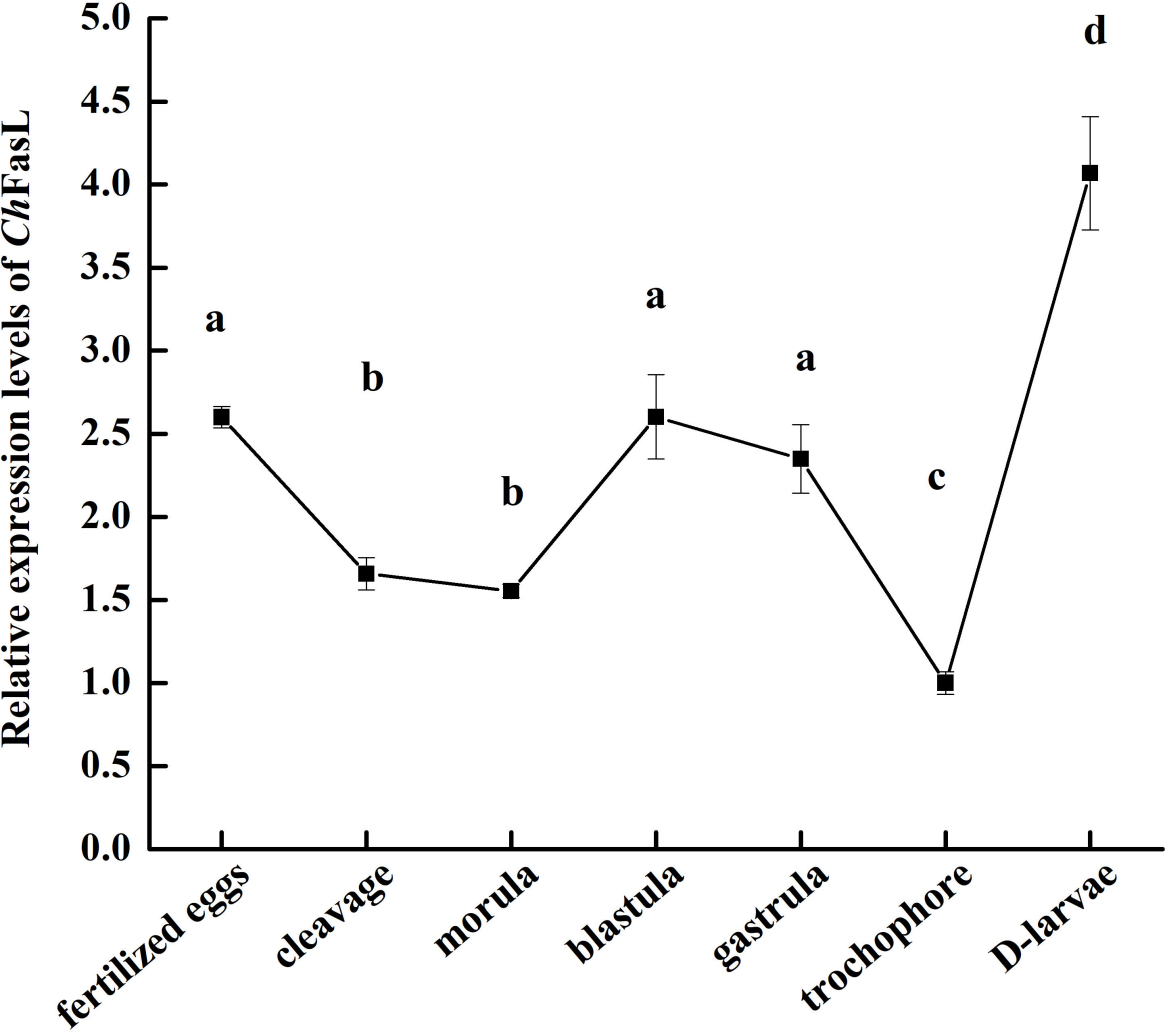
Real-time quantitative PCR analysis of *Ch*FasL in different larval development stages. Different letters indicate significant differences.

**Figure 4:**
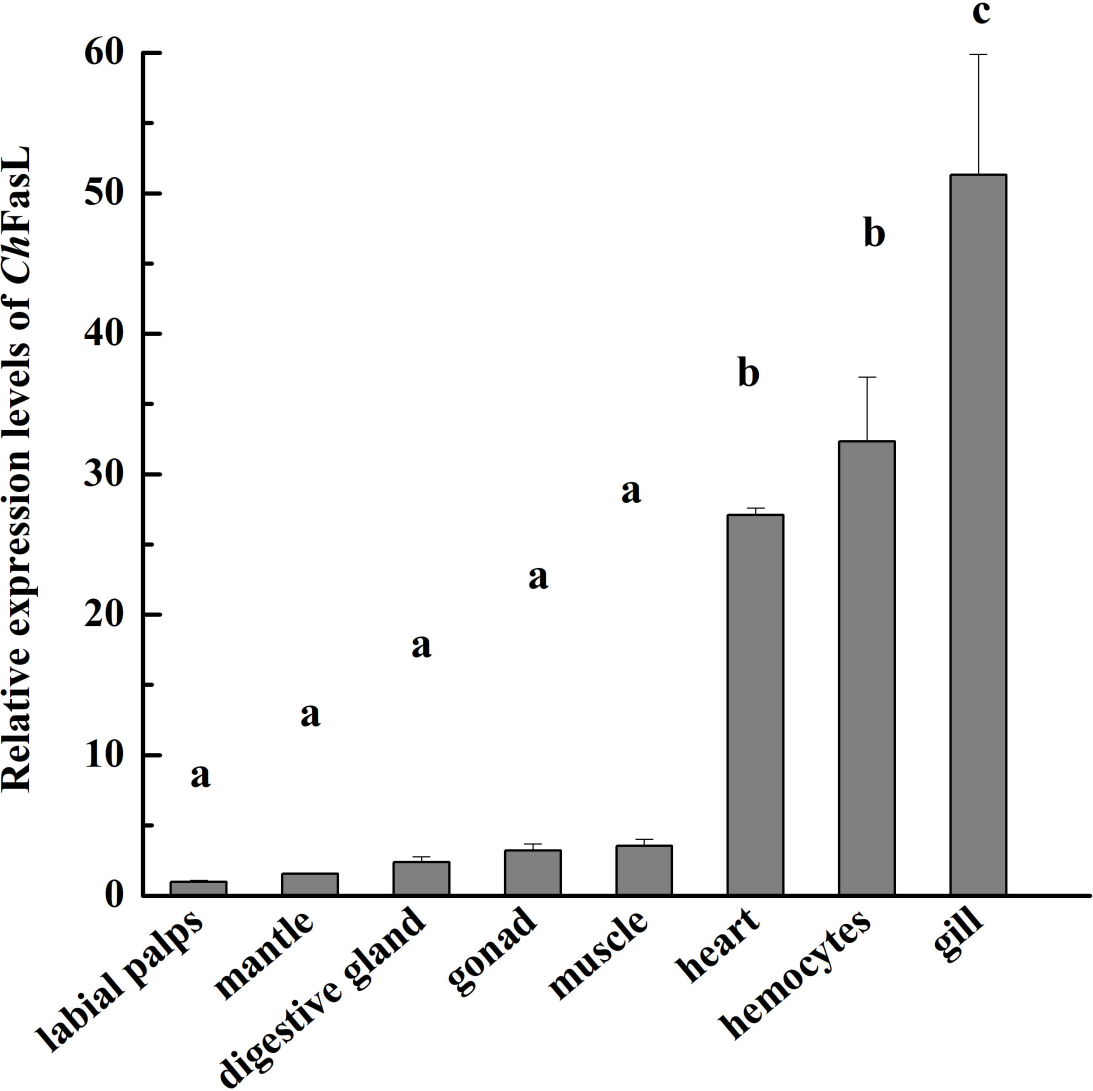
Real-time quantitative PCR analysis of *Ch*FasL in different tissues. Different letters indicate significant differences.

In order to understand the differences of *Ch*FasL expressions at different tissues and developmental stages, quantitative real-time PCR (qRT-PCR) was performed using primers specially designed according to the ORF sequence of *Ch*FasL gene. The relative expression of *Ch*FasL was detected throughout the whole development stages, with the highest expression in D-larvae stage and the lowest expression in trochophore stage, and the expression level in the D-larvae stage was 4.0-fold higher than in the trochophore stage (**Figure 3**). In addition, the results of qRT-PCR of different tissues showed that the relative expression of *Ch*FasL gene was visible in all 8 different tissues of Hong Kong oysters, and the expression level was significantly different, with the highest relative expression in the gills and the lowest expression level in the labial palps, and the relative expression of muscle was significantly different from that in hemocytes and heart. The relative expression level in the gill was about 1.59 times that in the hemocytes, 1.89 times that in the heart and 14.49 times that in the muscle (**Figure 4**).

**Figure 5:**
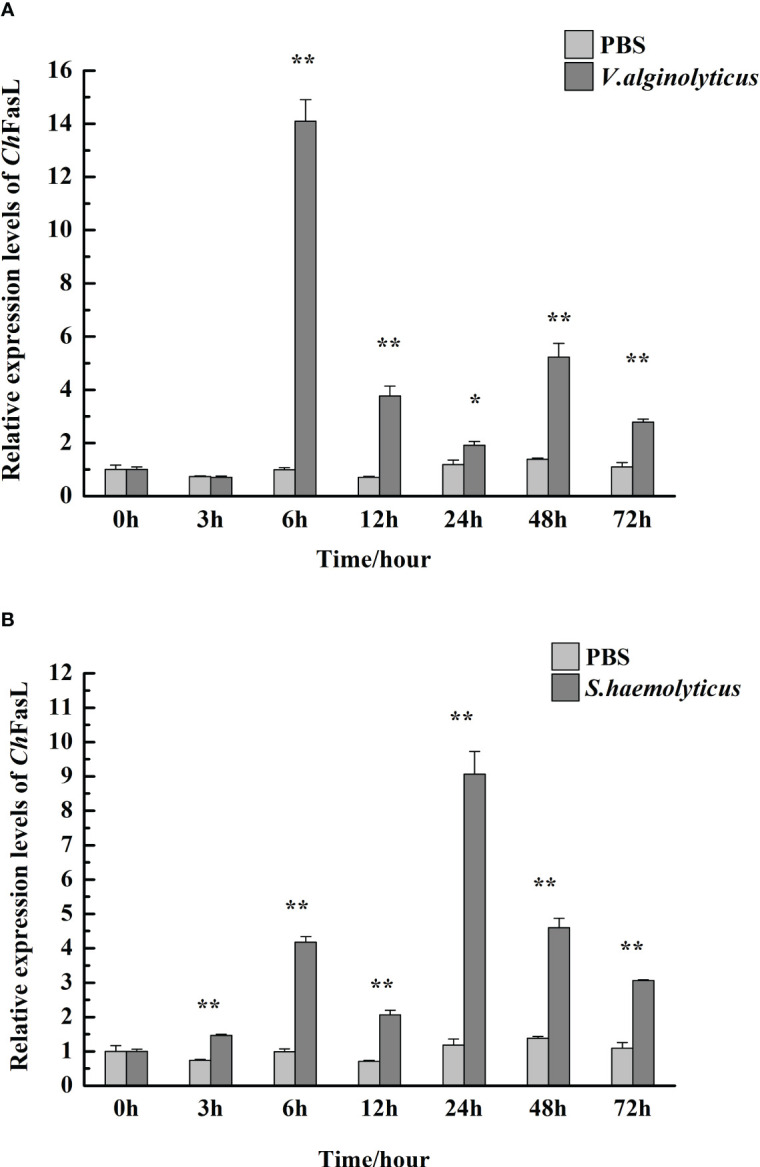
The relative mRNA expression of *Ch*FasL in hemocytes after injection with *V. alginolyticus* **(A)** and *S. haemolyticus* **(B)**. Significant differences are indicated by an asterisk (* and **, which represent *P*<0.01 and *P*<0.001, respectively).

After injection of *V. alginolyticus* and *S. haemolyticus*, the relative expressions of *Ch*FasL in hemocytes increased significantly (**Figure 5**). In the *V. alginolyticus* group, the relative expression of *Ch*FasL increased significantly and reached the peak at 6h after stimulation (*P* < 0.01), at which time the expression level was 14.09-fold higher than that of 0h. Although the relative expression of *Ch*FasL gradually decreased at 12h and 24h, it was still significantly higher than that of the PBS group (*P* < 0.01), and the expression level began to increase significantly again at 48h (*P* < 0.01, **Figure 5A**). However, in the *S. haemolyticus* group, the relative expression of *Ch*FasL increased significantly from the 3h and was significantly higher than that of the PBS control group (*P* < 0.01), but reached the peak at 24h after injection (*P* < 0.01), at which time the expression level was 9.07-fold higher than that of 0h, afterwards the relative expression level decreased steadily from 24h to 72h, but the relative expressions of *Ch*FasL were significantly higher than that of the PBS group (*P* < 0.05, **Figure 5B**).

### 3.4 Subcellular localization of *Ch*FasL and dual luciferase reporter gene assays

**Figure 6:**
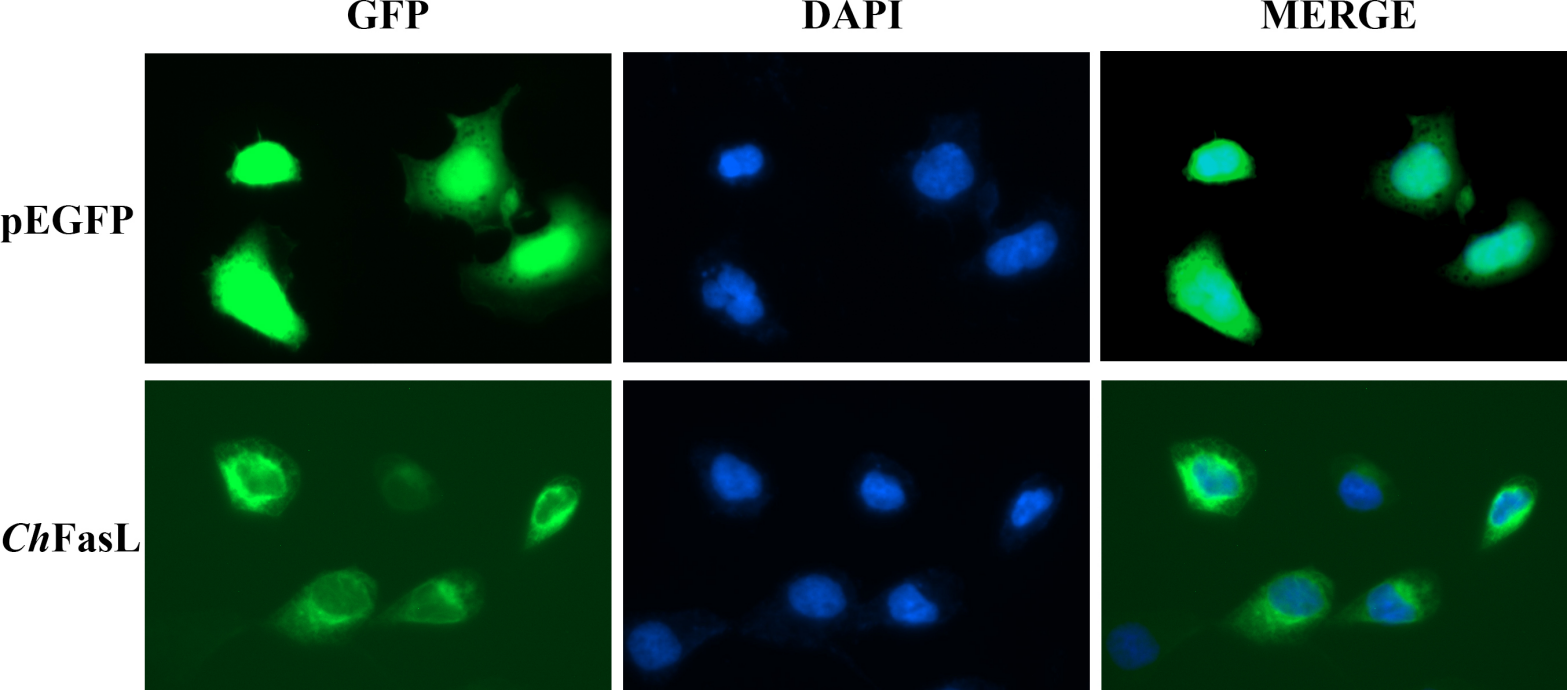
Subcellular localization analysis of *Ch*FasL in HEK293T cells. pEGFP-N1 was used as a control, and it was localized throughout the entire cell.

Subcellular localization of *Ch*FasL was performed by transferring pEGFP-N1- *Ch*FasL plasmid with green fluorescent protein expression into HEK293T cells, and then observing the location of green fluorescence expression by a fluorescence microscope, while pEGFP-N1 served as the control group. After 48 h of transfection, it was found that the green fluorescence expressed by the pEGFP-N1 plasmid was widely distributed throughout the cell, including the cytoplasm and nucleus, while the green fluorescence expressed by pEGFP-N1- *Ch*FasL plasmid was mainly expressed in the cytoplasm, indicating that the *Ch*FasL gene was localized in the cytoplasm (**Figure 6**).

**Figure 7:**
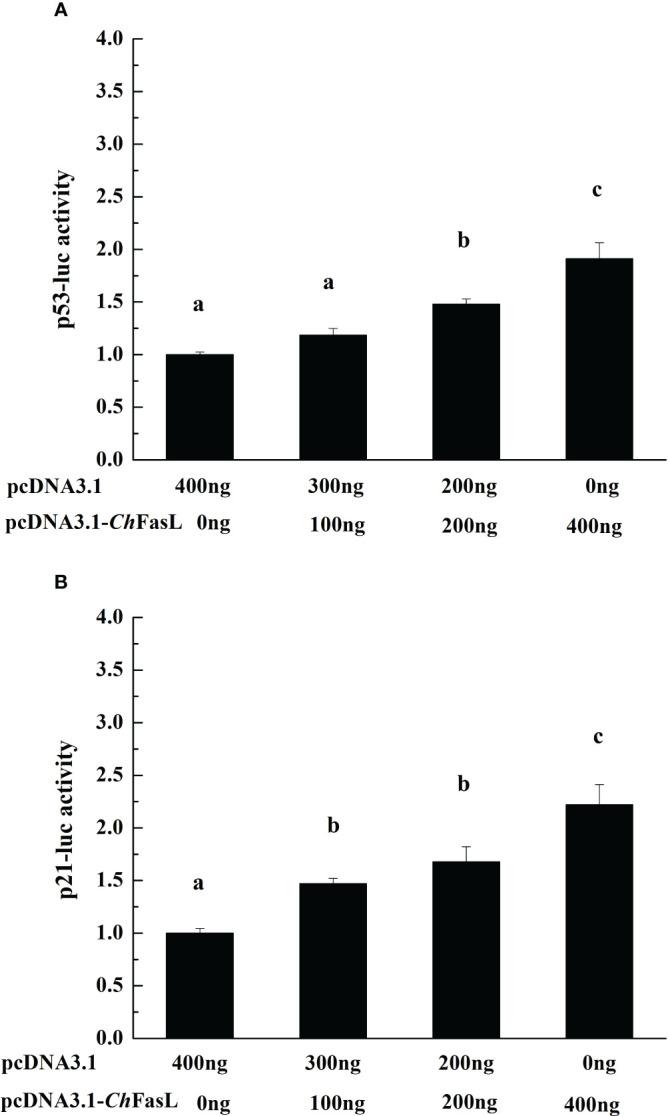
The change of reporter genes activities with the increase of recombinant protein pcDNA 3.1-*Ch*FasL content. **(A)** p53 and pcDNA 3.1-*Ch*FasL was simultaneously transferred to HEK293T cells. **(B)** p21 and pcDNA3.1- *Ch*FasL was simultaneously transferred into HEK293T cells. Different letters indicate significant differences.

In order to investigate the possible effects of *Ch*FasL on the activities of p53 and p21, a recombinant plasmid of pcDNA 3.1-*Ch*FasL was constructed and the dual luciferase reporter gene assays was performed. The results showed that overexpression of *Ch*FASL-related fusion proteins significantly promoted the activity of p53 luciferase activity in a dose-dependent manner. When the amount of recombinant plasmid increased to 300 ng or 400 ng, the activity of p53 could reach 1.48-fold or 1.91-fold higher (*P* < 0.05) than recombinant plasmid 0 ng (the negative control), respectively (**Figure 7A**). Similarly, overexpression of *Ch*FasL also significantly increased p21 activity, with the p21 activity in the 400 ng recombinant plasmid group being 2.22 times higher than that the negative control group, and significantly higher than the 200 ng and 100 ng recombinant plasmid groups (**Figure 7B**). These results suggested that *Ch*FasL is a positive transcription factor that regulates the activity of p53 and p21.

### 3.5 Effects of RNA interference of *Ch*FasL on cell apoptosis of oysters

**Figure 8:**
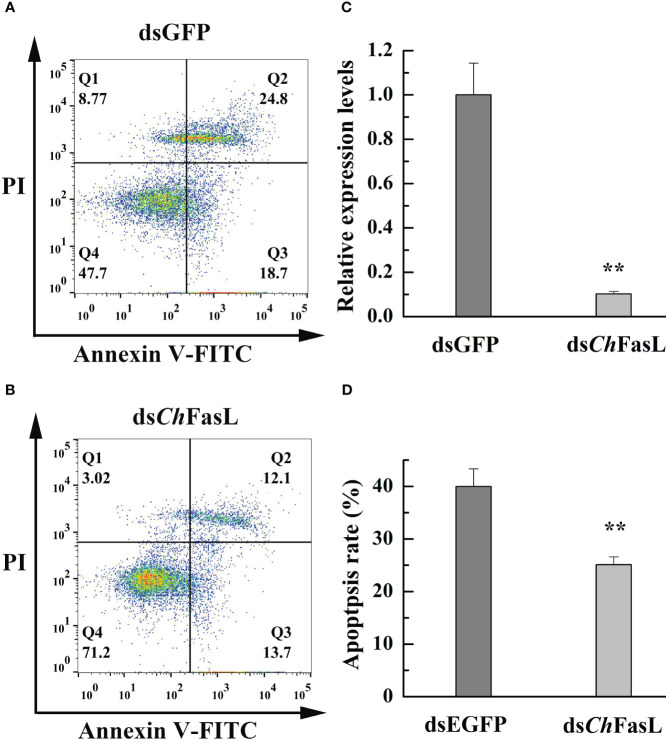
The change of apoptosis rate in the hemocytes of *C. hongkongensis* after *Ch*FasL RNAi (dsGFP as a control). **(A, B)** Representative images of flow cytometry analysis in haemocyte apoptosis after double staining of *C. hongkongensis* with Annexin-V-FITC and PI. Q1, Q2, Q3 and Q4 represent dead cells, late apoptotic cells, early apoptotic cells and living cells, respectively. **(C)** Relative expression of *Ch*FasL in hemocytes after RNAi. **(D)** The apoptosis rates in the hemocytes of *C. hongkongensis* after RNAi. ** represent *P*<0.01.

RNA interference (RNAi) was used to investigate the effect of *Ch*FasL on cell apoptosis. Compared with the dsGFP control group, the relative expression level of *Ch*FasL was significantly lower after 96 hours of double-RNA injection (*P* < 0.01), indicating that RNAi successfully interfered with the expression of *Ch*FasL gene (**Figure 8C**). In addition, flow cytometry showed that the apoptosis rate of ds*Ch*FasL group was significantly lower than that of dsGFP group (*P* < 0.05, **Figures 8A, B, D**).

## 4 Discussion

Invertebrates do not have an adaptive immune system, and only rely on the non-specific innate immune system composed of cellular and humoral immunity to eliminate invasive pathogens and maintain health (26). Apoptosis is an important part of the innate immune system of invertebrates. Apoptosis is a common physiological process, which can eliminate unwanted or diseased cells and play an important role in normal development, homeostasis, the formation of immune tolerance and response to environmental stress (16, 21). Apoptosis is also a physiologic protective mechanism that can removes unwanted, damaged or dangerous cells from the body without causing damage to surrounding cells (4, 27). The exogenous apoptotic pathway initiated by death receptors is regulated by multiple genes and factors, including Fas (APO-1 or CD95), Fas ligands (CD95 ligands), tumor necrosis factor (TNF), and related apoptosis-inducing ligands (Apo2L) (12, 15). It is well known that Fas and Fas ligand (FasL) systems play a central role in exogenous apoptosis. FasL, a type II membrane protein belonging to the TNF family, induces apoptosis by binding to Fas (5, 11). Despite the economic and ecological importance of bivalves, there is relatively little molecular information about Fas ligand genes or similar homologues in bivalves (16, 21, 28). Therefore, we report the first evidence of FasL in Hong Kong oyster, including its full-length characterization, expression profile, immune response, and validation of biological function.

The results of *Ch*FasL gene sequence cloning and comparison showed that *Ch*FasL has low homology with other vertebrate FasLs. Previous studies have pointed out that the FasL gene in aquatic organisms has low identity with that in mammals. For example, the amino acid identity between the FasL gene in Japanese flounder and human was only 26.1%, while the homology between FasL gene in abalone and human was 20.0%, and the FasL gene of *Ostrea edulis* also showed low identity, so it is normal for the *Ch*FasL gene to show low identity with other vertebrates (11, 21, 29–31). However, *Ch*FasL exhibits the same sequence characteristics as the FasL homologues, that is, the ORF has a transmembrane region, an N-glycosylation site, and a TNF region. The transmembrane region is essential for transmembrane protein, and the TNF region is a hallmark of the TNF family. A potential single N-glycosylation site has also been found in the abalone FasL, which usually exists in the extracellular portion of transmembrane protein (11, 31, 32). FasL of mammals and fishes often has a signal peptide, but it does not exist in *O. edulis* FasL and *Ch*FasL, and similar observation has been reported in abalone, so it is possible that bivalve FasL does not necessarily have signal peptide (10, 11). Based on the above characteristics, *Ch*FasL can be considered as a homolog of the Fas ligand in the TNF superfamily. In addition, the phylogenetic relationship analysis in this study is not very accurate, mainly due to the limited FasL sequences reported in lower invertebrate (5, 21).

The expressions of *Ch*FasL in different developmental stages and tissues of Hong Kong oyster was consistent with the findings in other aquatic organisms, mainly because although the FasL/Fas system has been studied and described in detail, it is mainly in hemolymph-mediated apoptosis. Many papers showed that Fas and FasL transcripts were also widely expressed and play a role in many tissues outside the immune system (11, 14, 21, 28, 33, 34). The expression of *Ch*FasL in tissues of Hong Kong oysters was the highest in the gills, followed by the hemocytes and heart, indicating that the expression pattern of *Ch*FasL in Hong Kong oysters was similar to that of FasL in abalone, which is also a mollusk, and the expression level of FasL in abalone gills was significantly higher than in other tissues (11, 35). The same expression pattern of FasL in Hong Kong oysters and abalone led us to speculate that FasL might have a common tissue expression pattern in shellfish, because their tissue function was not as specific as that of vertebrates (4, 8, 36). Apoptosis, including the FasL/Fas system, plays an important role in resistaning external environmental stimuli, microbial invasion, immunity and the elimination of unhealthy cells. The unique feeding patterns of shellfish lead to frequent close contact with pollutants, microorganisms and pathogens in external water, so FasL expression is highest in the gills of healthy individuals (9, 11, 30). As for the highest expression of FasL in the D larvae stage, it might be related to the fact that the larvae have completed hatching and began to resist external environmental changes and microbial invasion.

FasL is an important component of the exogenous pathway of apoptosis, mainly achieving its biological function by binding to the death receptor Fas on the cell surface, so *Ch*FasL like other organisms, is mainly localized in the cytoplasm (12, 14, 28, 34). It has been widely reported that apoptosis is an important mechanism for the protection of organisms, and the body uses apoptosis to eliminate cells damaged by environmental stresses or infected by pathogens (15, 19, 21). After infection by *V. alginolyticus* or *S. haemolyticus*, the expression of FasL gene in haemolymphs was increased significantly, accelerating cell apoptosis and immune response, and initiating the process of resistance to microbial infection. FasL is mainly involved in inflammatory and antimicrobial innate immune responses by the directly killing target cells by recruiting phagocytes, and also can be involved in immune responses by activating non-apoptotic signaling pathways (13, 19, 37, 38). After infection with *V. alginolyticus* or *S. haemolyticus*, the expression level of FasL increased, but with different expression profiles, indicating that although this gene participated in the immune response to bacterial infection, the response time and degree to different infection sources were different.

The process of apoptosis is strictly controlled by signals, and factors that regulate apoptosis include anti-apoptotic factors and pro-apoptotic factors. Therefore, the fate of cells: survival or death, may depend on the relative content of these two types of regulatory factors in the cells and the regulation of their activity by extracellular signals (4, 9, 37). p53 and p21 are important pro-apoptotic factors in mammals, which together constitute the G1 checkpoint of the cell cycle, which cannot pass without repair after DNA damage, reducing the replication and accumulation of damaged DNA, and thus playing a role in cancer inhibition (6, 26, 39). Under normal circumstances, the activity of p53 in cells is kept at a very low level, and p53 will be activated when cells encounter abnormal conditions such as DNA damage, then cell growth will be stopped by blocking the cell cycle, and then apoptosis will be triggered (39, 40). In the process of p53-dependent apoptosis, p53 promotes apoptosis by activating the transcription of positive regulatory factors of apoptosis, such as DR5, Bax, and p53-inducible gene. In addition, p53 can inhibit the transcription of anti-apoptotic factors, such as Bcl-2 and Survivin (12, 15, 33, 39). In molluscs, p53 protein has significant sequence and structural similarities with human p53, and is localized in the nucleus in response to apoptosis signals (26, 39). The overexpression of mortalins (HSP70 family proteins), which bind to p53, inhibits the expression of pro-apoptotic p53-dependent genes, and reduces the level of apoptosis in mollusk cells in a similar manner to that of vertebrates (9, 10, 39). In *C. gigas*, *O. edulis* and *H. discus discus*, p53 was found to activate pro-apoptotic genes and induce apoptosis of oyster cells (11, 21, 41). Therefore, the function of mollusk p53 and mammalian p53 should be similar. Our experimental results showed that the activities of p53 and p21 were significantly increased with the increase of recombinant protein pcDNA3.1-*Ch*FasL content, showing a positive regulatory effect, which also indicated that they were two important pro-apoptotic factors in Hong Kong oysters. FasL plays a very important role in mammalian apoptosis, but its studies in molluscs is limited, with only shallow studies in *O. edulis* and *H. discus discus* (11, 21). In order to further prove the relationship between *Ch*FasL and apoptosis in *C. hongkongensis*, RNAi experiments were conducted. After dsRNA successful reduced the expression of *Ch*FasL the apoptosis rate of haemolymphs was significantly lower than that of the control group, which can serve as another direct evidence that *Ch*FasL was involved in the apoptosis of *C. hongkongensis*. This finding is consistent with previous research on other key apoptotic genes, and silencing apoptotic genes can lead to a decrease in apoptosis (16, 24, 26).

In conclusion, we cloned a FasL gene from *C. hongkongensis* for the first time and named it *Ch*FasL. We found that *Ch*FasL has typical sequence characteristics of the Tumor necrosis factor family, including potential N-linked glycosylation site, a transmembrane region and a TNF region. Quantitative expression analysis in different developmental stages and tissues showed that *Ch*FasL was expressed in all developmental stages and tissues, and the relative expression of *Ch*FasL was significantly increased after infected by *V. alginolyticus* or *S. haemol*yticus, suggesting that *Ch*FasL may involve in the immune response of Hong Kong oyster to pathogenic microbial infection. In addition, *Ch*FasL was mainly distributed in the cytoplasm of HEK293T, and could positively regulate the activities of p53 and p21. Combined with the results of decreasing the apoptosis rate after RNA interference of *Ch*FasL expression, it showed that *Ch*FasL was deeply involved in the apoptosis process of Hong Kong oyster. Overall, this study demonstrates that *Ch*FasL plays an important role in apoptosis and immune response, similar to FasL homologues in other organisms. This study provides a basis and premise for further understanding the process of apoptosis and non-specific innate immunity in mollusks, and also contributes to further research on the pathogenesis and disease resistance breeding of *C. hongkongensis*.

## Data availability statement

The data presented in the study are deposited in the NCBI GenBank repository, accession number OR157977.

## Ethics statement

The studies involving animals were reviewed and approved by the Animal Care and Ethics Committee of South China Sea Institute of Oceanology, Chinese Academy of Sciences.

## Author contributions

YQ: Data curation, Formal Analysis, Funding acquisition, Investigation, Resources, Software, Supervision, Visualization, Writing – original draft, Writing – review & editing. WW: Investigation, Formal Analysis, Resources, Software. JWL: Formal Analysis, Investigation, Supervision. ZW: Formal Analysis, Investigation. YY: Software, Conceptualization, Data curation, Formal Analysis, Investigation. JL: Investigation, Software. HM: Methodology. ZY: Writing – review & editing, Funding acquisition, Visualization. ZX: Investigation, Supervision, Visualization, Writing – review & editing. YZ: Funding acquisition, Investigation, Resources, Supervision, Validation, Visualization, Writing – review & editing.

## References

[1] Tittel JN Steller H. A comparison of programmed cell death between species. Genome Biol (2000) 1(3):reviews0003. 1. doi: 10.1186/gb-2000-1-3-reviews0003 11178240 PMC138857

[2] Kerr JF Wyllie AH Currie AR. Apoptosis: A basic biological phenomenon with wideranging implications in tissue kinetics. Br J Cancer (1972) 26(4):239. doi: 10.1038/bjc.1972.33 4561027 PMC2008650

[3] Reed JC. Mechanisms of apoptosis. Am J Pathol (2000) 157(5):1415–30. doi: 10.1016/S0002-9440(10)64779-7 PMC1885741 11073801

[4] Hanayama R Tanaka M Miwa K Shinohara A Iwamatsu A Nagata S. Identification of a factor that links apoptotic cells to phagocytes. Nature (2002) 417(6885):182. doi: 10.1038/417182a 12000961

[5] Hengartner MO. The biochemistry of apoptosis. Nature (2000) 407(6805):770. doi: 10.1038/35037710 11048727

[6] Edinger AL Thompson CB. Death by design: apoptosis, necrosis and autophagy. Curr Opin Cell Biol (2004) 16(6):663–9. doi: 10.1016/j.ceb.2004.09.011 15530778

[7] Ellis RE Yuan J Horvitz HR. Mechanisms and functions of cell death. Annu Rev Cell Biol (1991) 7(1):663–98. doi: 10.1146/annurev.cb.07.110191.003311 1809356

[8] Ellis HM Horvitz HR. Genetic control of programmed cell death in the nematode C. elegans. Cell (1986) 44(6):817–29. doi: 10.1016/0092-8674(86)90004-8 3955651

[9] Elmore S. Apoptosis: A review of programmed cell death. Toxicol Pathol (2007) 35(4):495–516. doi: 10.1080/01926230701320337 17562483 PMC2117903

[10] Opferman JT Korsmeyer SJ. Apoptosis in the development and maintenance of the immune system. Nat Immunol (2003) 4(5):410. doi: 10.1038/ni0503-410 12719730

[11] De Zoysa M Nikapitiya C Moon D-O Whang I Kim G-Y Lee J. A novel fas ligand in mollusk abalone: molecular characterization, immune responses and biological activity of the recombinant protein. Fish Shellfish Immunol (2009) 27(3):423–32. doi: 10.1016/j.fsi.2009.06.019 19576285

[12] Fuchs Y Steller H. Programmed cell death in animal development and disease. Cell (2011) 147(4):742–58. doi: 10.1016/j.cell.2011.10.033 PMC4511103 22078876

[13] Carson DA Ribeiro JM. Apoptosis and disease. Lancet (1993) 341(8855):1251–4. doi: 10.1016/0140-6736(93)91154-E 8098400

[14] Nagata S. Fas ligand-induced apoptosis. Annu Rev Genet (1999) 33(1):29–55. doi: 10.1146/annurev.genet.33.1.29 10690403

[15] Wajant H. The fas signaling pathway: more than a paradigm. Science (2002) 296(5573):1635–6. doi: 10.1126/science.1071553 12040174

[16] Zhang L Li L Zhang G. Gene discovery, comparative analysis and expression profile reveal the complexity of the *Crassostrea gigas* apoptosis system. Dev Comp Immunol (2011) 35(5):603–10. doi: 10.1016/j.dci.2011.01.005 21237195

[17] Park HH Lo Y Lin S Wang L Yang JK Wu H. The death domain superfamily in intracellular signaling of apoptosis and inflammation. Annu Rev Immunol (2007) 25:561–86. doi: 10.1146/annurev.immunol.25.022106.141656 PMC2904440 17201679

[18] Cohen GM. Caspases: the executioners of apoptosis. Biochem J (1997) 326(Pt 1):1. doi: 10.1042/bj3260001 9337844 PMC1218630

[19] Shi Y. Mechanisms of caspase activation and inhibition during apoptosis. Mol Cell (2002) 9(3):459–70. doi: 10.1016/S1097-2765(02)00482-3 11931755

[20] Dianzani U Bragardo M DiFranco D Alliaudi C Scagni P Buonfiglio D. Deficiency of the fas apoptosis pathway without fas gene mutations in pediatric patients with autoimmunity/lymphoproliferation. Blood (1997) 89(8):2871–9. doi: 10.1182/blood.V89.8.2871 9108407

[21] Morga B Renault T Faury N Arzul I. New insights in flat oyster Ostrea edulis resistance against the parasite bonamia ostreae. Fish Shellfish Immunol (2012) 32(6):958–68. doi: 10.1016/j.fsi.2012.01.026 22406616

[22] Cuesta A Esteban MA Meseguer J. Identification of a fasl-like molecule in leucocytes of the teleost fish gilthead seabream (Sparus aurata L.). Dev Comp Immunol (2003) 27(1):21–7. doi: 10.1016/S0145-305X(02)00041-1 12477498

[23] Killen SS Costa I Brown JA Gamperl AK. Little left in the tank: metabolic scaling in marine teleosts and its implications for aerobic scope. Proc R Soc B: Biol Sci (2006) 274(1608):431–8. doi: 10.1098/rspb.2006.3741 PMC1702384 17164208

[24] Kurobe T Hirono I Kondo H Saito-Taki T Aoki T. Molecular cloning, characterization, expression and functional analysis of Japanese flounder *Paralichthys olivaceus* fas ligand. Dev Comp Immunol (2007) 31(7):687–95. doi: 10.1016/j.dci.2006.08.006 17197025

[25] Qin Y Zhang Y Mo R Zhang Y Li J Zhou Y. Influence of ploidy and environment on grow-out traits of diploid and triploid Hong Kong oysters *Crassostrea hongkongensis* in Southern China. Aquaculture (2019) 507:108–18. doi: 10.1016/j.aquaculture.2019.04.017

[26] Qin Y Zhang Y Li X Noor Z Li J Zhou Z. Characterization and functional analysis of a caspase 3 gene: evidence that chcas 3 participates in the regulation of apoptosis in *Crassostrea hongkongensis* . Fish Shellfish Immunol (2020) 98:122–9. doi: 10.1016/j.fsi.2020.01.007 31917320

[27] Witkop EM Proestou DA Gomez-Chiarri M. The expanded inhibitor of apoptosis gene family in oysters possesses novel domain architectures and may play diverse roles in apoptosis following immune challenge. BMC Genomics (2022) 23(1):1–31. doi: 10.1186/s12864-021-08233-6 35279090 PMC8917759

[28] Romero A Estevez-Calvar N Dios S Figueras A Novoa B. New insights into the apoptotic process in mollusks: characterization of caspase genes in Mytilus galloprovincialis. PLos one (2011) 6(2):e17003. doi: 10.1371/journal.pone.0017003 21347300 PMC3037946

[29] Goetz FW Planas JV MacKenzie S. Tumor necrosis factors. Dev Comp Immunol (2004) 28(5):487–97. doi: 10.1016/j.dci.2003.09.008 15062645

[30] Schultz U Kaspers B Staeheli P. The interferon system of non-mammalian vertebrates. Dev Comp Immunol (2004) 28(5):499–508. doi: 10.1016/j.dci.2003.09.009 15062646

[31] Hirono I Nam B-H Kurobe T Aoki T. Molecular cloning, characterization, and expression of tnf cdna and gene from Japanese flounder *Paralychthys olivaceus* . J Immunol (2000) 165(8):4423–7. doi: 10.4049/jimmunol.165.8.4423 11035080

[32] Spiro RG. Protein glycosylation: nature, distribution, enzymatic formation, and disease implications of glycopeptide bonds. Glycobiology (2002) 12(4):43R–56R. doi: 10.1093/glycob/12.4.43R 12042244

[33] Kono T Zou J Bird S Savan R Sakai M Secombes CJ. Identification and expression analysis of lymphotoxin-beta like homologues in rainbow trout *Oncorhynchus mykiss* . Mol Immunol (2006) 43(9):1390–401. doi: 10.1016/j.molimm.2005.07.037 16144708

[34] Liu B Li G Yang J Li X Wang H Yang J. The mechanism of immune related signal pathway egr2-fasl-fas in transcription regulation and methylated modification of *Paralichthys olivaceus* under acute hypoxia stress. Fish Shellfish Immunol (2022) 123:152–63. doi: 10.1016/j.fsi.2022.02.036 35219829

[35] De Zoysa M Jung S Lee J. First molluscan Tnf-A Homologue of the Tnf superfamily in disk abalone: molecular characterization and expression analysis. Fish Shellfish Immunol (2009) 26(4):625–31. doi: 10.1016/j.fsi.2008.10.004 18984056

[36] Ma T Wu J Gao X Wang J Zhan X Li W. Molecular cloning, functional identification and expressional analyses of fasl in tilapia, *Oreochromis niloticus* . Dev Comp Immunol (2014) 46(2):448–60. doi: 10.1016/j.dci.2014.06.003 24950416

[37] Wajant H Pfizenmaier K Scheurich P. Non-apoptotic fas signaling. Cytokine Growth Factor Rev (2003) 14(1):53–66. doi: 10.1016/S1359-6101(02)00072-2 12485619

[38] Ahn JH Park SM Cho HS Lee MS Yoon JB Vilcek J. Non-apoptotic signaling pathways activated by soluble fas ligand in serum-starved human fibroblasts: mitogen-activated protein kinases and Nf-Kb-dependent gene expression. J Biol Chem (2001) 276(50):47100–6. doi: 10.1074/jbc.M107385200 11600497

[39] Sokolova I. Apoptosis in molluscan immune defense. Invertebrate Survival J (2009) 6(1):49–58. Available at: https://www.isj.unimore.it/index.php/ISJ/article/view/179.

[40] Zacks DN Kocab AJ Choi JJ Gregory-Ksander MS Cano M Handa JT. Cell death in amd: the rationale for targeting fas. J Clin Med (2022) 11(3):592. doi: 10.3390/jcm11030592 35160044 PMC8836408

[41] Li Y Zhang L Qu T Tang X Li L Zhang G. Conservation and divergence of mitochondrial apoptosis pathway in the pacific oyster, *Crassostrea gigas* . Cell Death Dis (2017) 8(7):e2915. doi: 10.1038/cddis.2017.307 28682310 PMC5550854

